# Resistance to Sri Lankan Cassava Mosaic Virus (SLCMV) in Genetically Engineered Cassava cv. KU50 through RNA Silencing

**DOI:** 10.1371/journal.pone.0120551

**Published:** 2015-04-22

**Authors:** Valentine Otang Ntui, Kynet Kong, Raham Sher Khan, Tomoko Igawa, Gnanaguru Janaky Janavi, Ramalingam Rabindran, Ikuo Nakamura, Masahiro Mii

**Affiliations:** 1 Laboratory of Plant Cell Technology, Graduate School of Horticulture, Chiba University, Chiba, Japan; 2 Department of Genetics/Biotechnology, Faculty of Science, University of Calabar, Calabar, Nigeria; 3 Cambodia Agricultural Research and Development Institute, Phnom Penh, Cambodia; 4 Department of Biotechnology, Abdul Wali Khan University, Mardan, Pakistan; 5 Horticultural College and Research Institute, Tamil Nadu Agricultural University, Coimbatore, India; Shanghai Institutes for Biological Sciences, CHINA

## Abstract

Cassava ranks fifth among the starch producing crops of the world, its annual bioethanol yield is higher than for any other crop. Cassava cultivar KU50, the most widely grown cultivar for non-food purposes is susceptible to Sri Lankan cassava mosaic virus (SLCMV). The objective of this work was to engineer resistance to SLCMV by RNA interference (RNAi) in order to increase biomass yield, an important aspect for bioethanol production. Here, we produced transgenic KU50 lines expressing dsRNA homologous to the region between the AV2 and AV1 of DNA A of SLCMV. High level expression of dsRNA of SLCMV did not induce any growth abnormality in the transgenic plants. Transgenic lines displayed high levels of resistance to SLCMV compared to the wild-type plants and no virus load could be detected in uninoculated new leaves of the infected resistant lines after PCR amplification and RT-PCR analysis. The agronomic performance of the transgenic lines was unimpaired after inoculation with the virus as the plants presented similar growth when compared to the mock inoculated control plants and revealed no apparent reduction in the amount and weight of tubers produced. We show that the resistance is correlated with post-transcriptional gene silencing because of the production of transgene specific siRNA. The results demonstrate that transgenic lines exhibited high levels of resistance to SLCMV. This resistance coupled with the desirable yield components in the transgenic lines makes them better candidates for exploitation in the production of biomass as well as bioethanol.

## Introduction

Cassava (*Manihot esculenta*) is an important root crop in Africa, Asia and South America, providing energy for about one billion people. It is the 3^rd^ largest source of carbohydrates for human food in the world, a staple or subsidiary food for about a fifth of the world’s population and raw material for starch based industries. Its flexible harvesting time, tolerance to adverse environmental conditions such as drought and poor soils, little requirement to agricultural fertilizers and high starch content make cassava one of the most attractive plants for starch production in future. In recent years, cassava has emerged as a potential biofuel plant, ranking among other high energy crops such as maize, sugarcane and sweet sorghum. Indeed, the annual yield of cassava-derived bioethanol was found to be higher than for any other crop, including sugarcane [1–3], and its bioethanol production has been increasing due to its economic benefits compared to other bioethanol production crops. Unfortunately, cassava production is being increasingly affected by cassava mosaic disease (CMD), resulting to losses estimated at 1.6 billion dollars [4].

CMD is caused by several cassava mosaic begomoviruses, which are transmitted, plant-to-plant, exclusively by the whitefly *Bemisia tabaci*. The cassava bipartite begomovirus genome comprises two circular, single-stranded DNA components, which are DNA A and DNA B, and possess a segment of high sequence identity (∼200 nucleotides in length) known as the common region (CR). The CR harbours the viral promoters, origin of replication and sequences involved in binding of DNA A-encoded replication associated protein (Rep), the only virus encoded product required for viral DNA replication [5]. DNA A encodes two overlapping virion-sense open reading frames (ORFs) AV2 and AV1, and at least four overlapping complementary-sense ORFs AC1, AC2, AC3 and AC4. These genes are involved in encapsidation, viral DNA replication and control of gene expression. AV1 encodes the coat protein gene (CP) and is the determinant of vector transmission in addition to its role in genome encapsidation. AC1 encodes a replication-associated protein (Rep), AC2 a transcriptional activator protein (TrAP) and also functions in the suppression of post-transcriptional gene silencing, and AC3 a replication enhancer protein (REn). AC4 plays a role as a suppressor of RNA silencing. DNA B encodes two gene products, BV1 and BC1, which encode the nuclear shuttle protein and the movement protein, respectively [6].

CMD is widespread in Africa and the Indian subcontinent [7]. *African cassava mosaic virus* (ACMV) was the first virus species found to be associated with CMD in Africa, although no fewer than seven begomovirus species are now recognized in Africa [8]. In the Indian subcontinent, Indian cassava mosaic virus (ICMV) has been shown to be associated with CMD [9]. Another species, Sri Lankan cassava mosaic virus (SLCMV), was identified in Sri Lanka and clearly linked to cassava mosaic disease in India [10]. ICMV and SLCMV are distinct among the viruses associated with CMD in Africa and are reported to cause serious cassava infection in India. Survey of cassava mosaic disease in India revealed that SLCMV is the most prevalent virus [11].

To date, a number of strategies to engineer CMD resistance in cassava have been reported mostly for ACMV [12]. For example, increased ACMV resistance in cassava has been developed in transgenic cassava plants expressing antisense RNA or dsRNA targeting the viral mRNAs of Rep (AC1), TrAP (AC2) and REn (AC3), or the viral untranslational common region [13–15]. Until now, no such report exists on the production of transgenic cassava plants resistant to SLCMV. SLCMV has recently become a major concern and is rapidly emerging as an important CMD prevalent in the Indian subcontinent, resulting in serious yield losses [11].

CMD is currently managed by multiplication and distribution of disease-free stem cuttings. It has been difficult to produce SLCMV-resistant cassava by conventional breeding because of high heterozygosity and inbreeding depression of elite cultivars or farmer-preferred landraces. As a result, new strategies to control SLCMV are desirable. Post-transcriptional gene silencing (PTGS) or RNA interference (RNAi) is a technology that offers significant potential to control plant viral pathogens. RNAi has been applied to generate resistance to *African cassava mosaic virus* [15], *Cucumber mosaic virus*, *Zucchini yellow mosaic virus* and *Watermelon mosaic virus* [16–19], *Bean golden mosaic virus* [20], *Potato leaf roll virus*, *Potato virus Y and Potato virus X* [21, 22], *Papaya ring spot virus* [23] and *Plum pox virus* [24, 25].

RNA interference (RNAi) is a conserved mechanism that recognizes double-stranded RNA (dsRNA) as a signal to trigger sequence-specific degradation of homologous mRNA. The key feature of RNAi is short dsRNA fragments known as “short interfering RNAs (siRNAs)” of 21–25 bp in length, which are produced by the cleavage of dsRNA by a ds-specific ribonuclease termed ‘Dicer’. Once generated, the siRNA are then recognized by a ribonuclease complex known as the RNA-induced silencing complex (RISC) and used as a guide for the recognition and sequence-specific degradation of homologous mRNAs [26–29], resulting in post-transcriptional gene silencing (PTGS).

Currently, the elite cassava variety, Thai cassava cultivar Kasetsart University 50 (KU50) one of the most important cassava cultivar in the world is grown by many farmers and biofuel industries in Asia under different names because of its high root yield and high root starch content with good germination and vigorous plant growth with wide adaptation [30]. Therefore, evaluation of its capacity for SLCMV resistance is of importance due to devastating impact of this virus on cassava production in the region. Unfortunately, engineering disease resistance in this cultivar has faced a major setback and difficulties largely due to lack of efficient and reproducible regeneration protocol. An important prerequisite for engineering plants for resistance to diseases is the availability of morphogenic culture that can be used in gene transfer techniques [31].

In this article, we report a simple, efficient, quick and reproducible regeneration protocol for cassava cultivar KU50 through somatic embryogenesis. This protocol allowed us to produce transgenic cassava lines that express dsRNA homologous to the region between the AV2 and AV1 of SLCMV. Transgenic lines obtained displayed high levels of resistance to SLCMV and agronomic performance in the transgenic lines was not affected in the presence of the virus. We show that the resistance is correlated with post-transcriptional gene silencing because of the production of transgenic specific siRNA.

## Results

### Plant regeneration in cassava

**Fig 1:**
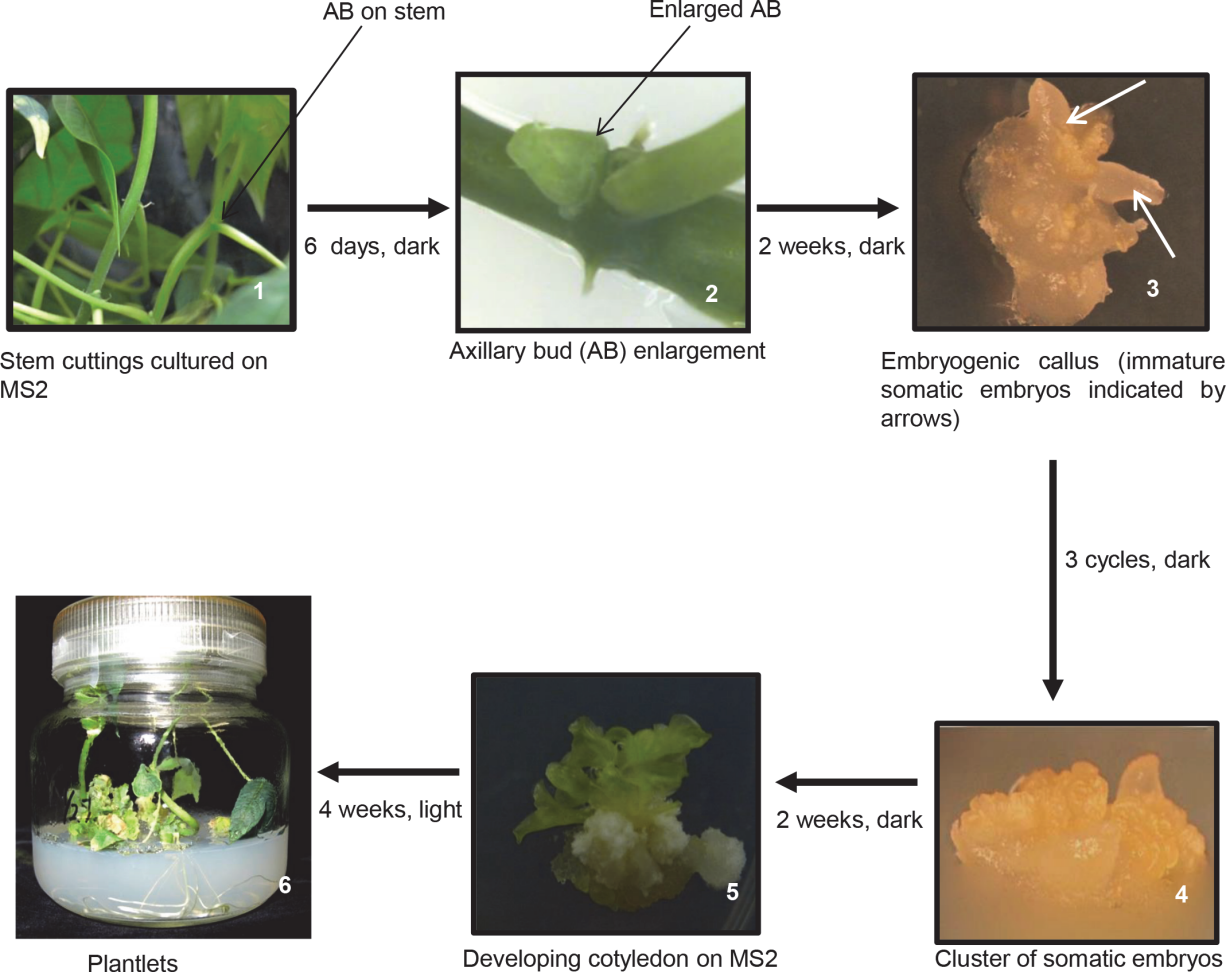
Main stages of SE production and plant regeneration in cassava cultivar KU50.

**Fig 2:**
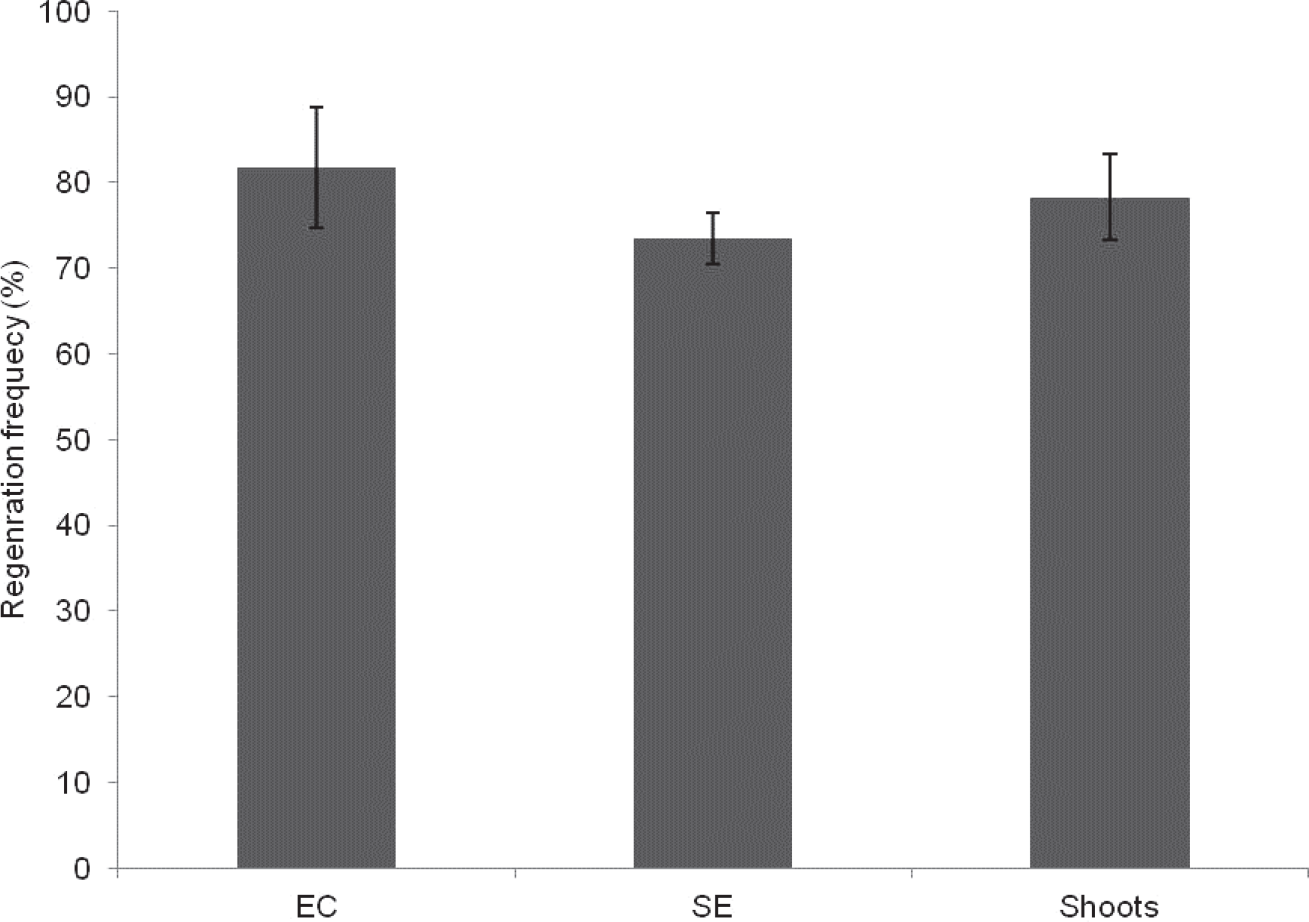
Number of embryos produced and plants regenerated from embryogenic lines of cassava cultivar KU50. EC, Embryogenic callus. SE, Somatic embryos

Enlarged axillary buds (Fig. 1, plate 2) were used for somatic embryo production and plant regeneration. When these were cultured on MSP, callus started 7 days after culture. Initially, most of the calli formed were soft and translucent (data not shown). After 2 weeks in culture on MSP medium in the dark, somatic embryos (SE), which had a globular appearance, were formed. Initially, the globular structures could not be separated easily from the originating tissue and the structures were fragile. Nevertheless, after additional 5 days in culture, distinct globular structures which closely resemble globular embryos in appearance were produced (Fig. 1, plate 3). After 3 cycles of transfer of SE in the same medium at 2 weeks interval with constant removal of soft non embyogenic tissues, somatic embryos that developed torpedo stage were isolated from mother tissues and transferred to fresh medium of the same composition for further growth (Fig. 1, plate 4). After 4 weeks, the somatic embryos were transferred to MS2 supplemented with 0.5 mg/l BA and 0.5 mg/l IBA. Cotyledons appeared after 2 weeks of culture (Fig. 1, plate 5). The cotyledons thus produced were transferred to hormone free MS medium and exposed to light for shoot and root development. After 4 weeks of culture under light condition, shoots and roots were produced (Fig. 1, plate 6). A summary of the main events is shown in Fig. 1. Also, the percentage of somatic embryos that produced shoots is shown in Fig. 2.

### Production of transgenic plants resistant to SLCMV

#### RNAi construct and cassava transformation

**Fig 3:**
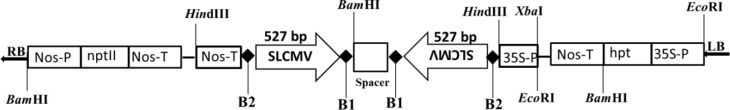
Map of T-DNA fragment of pEKH2IN2 carrying Sri Lankan cassava mosaic virus (SLCMV) inverted repeat. The SLCMV gene and hygromycin phosphotransferase (hpt) are driven by CaMV 35S promoter (35SP), and the gene for neomycin phosphotransferase (nptII) by nopaline synthase promoter (nos-p). B1 and B2 represent attB1 and attB2 recombination sites, respectively; LB and RB, left and right borders of T-DNA sequences, respectively; nos-T, terminator of the nopaline synthase gene. Recognition sites of restriction enzymes are also indicated.

In order to express virus-derived dsRNA in cassava, we produced a construct containing inverted repeat of 527 bp DNA fragment of SLCMV. The 527 bp fragment, which includes part of the AV2 (137–493 bp) and half of the AV1 (297–534 bp) spans from nucleotide 8–534. To ensure stability of the inverted repeat in *Escherichia coli*, the two DNA fragments were separated by a 278 bp cat 1 1^st^ intron spacer derived from castor bean catalase gene [32]. The entire cassette was cloned in the plant transformation vector, pEKH2IN2 yielding pEKH2IN2SLCMV (Fig. 3), with the SLCMV gene being driven by cauliflower mosaic virus (CaMV) 35S promoter.

**Fig 4:**
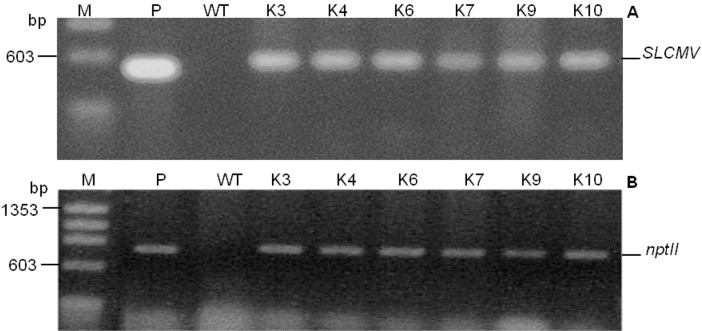
Detection of 527 bp *SLCMV* and 700 bp *nptII* genes in transgenic cassava lines. Lane M, molecular size marker (ø174HaeIII digests). Lane P, positive control (plasmid DNA). Lane WT, non-transformed wild-type control plant. Lanes K3–K10, independent transgenic cassava lines.

**Fig 5:**
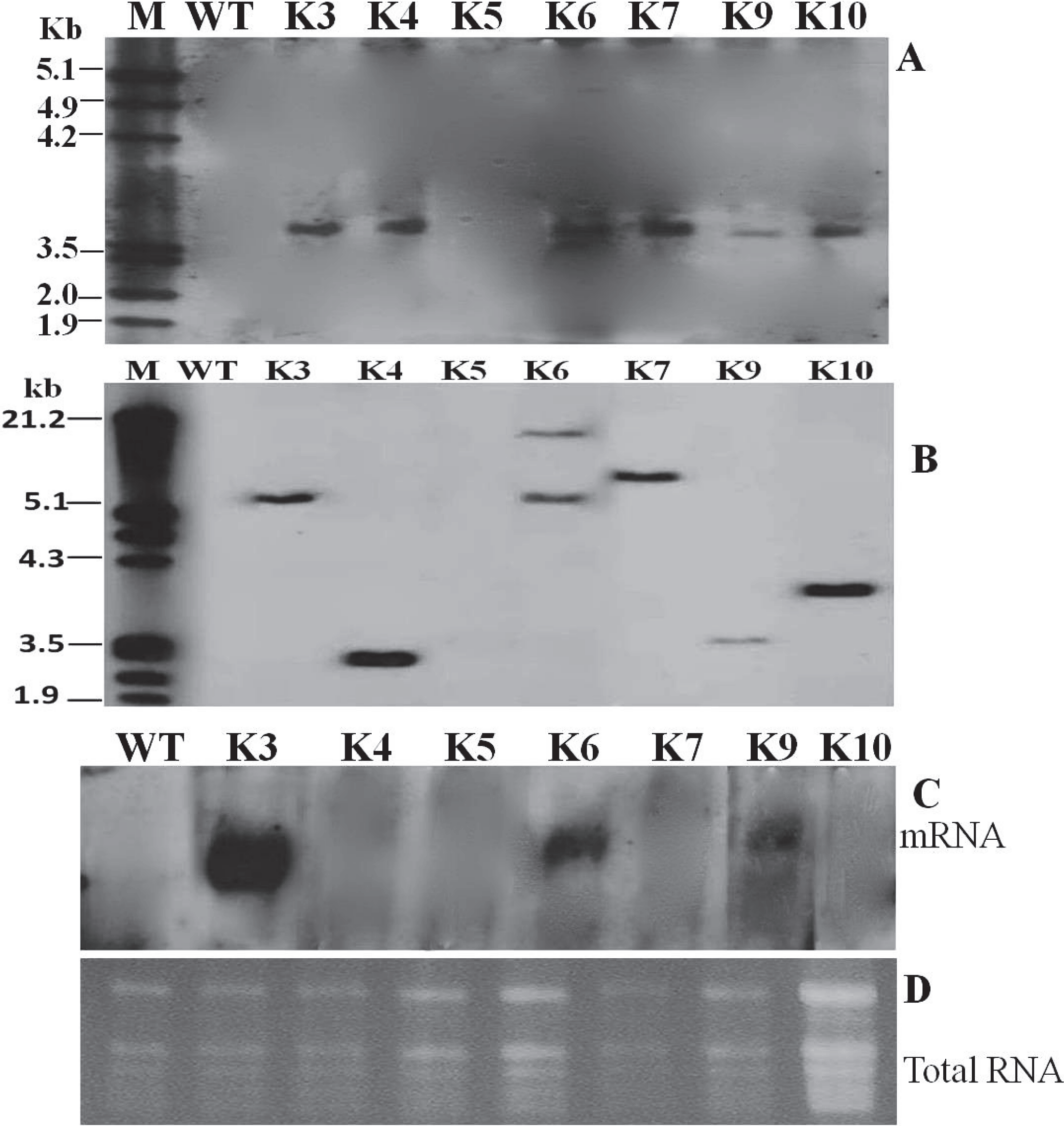
Molecular analysis of transgenic cassava lines expressing hairpin RNA of SLCMV AV2::AV1. Southern blot analysis of genomic DNA from transgenic and non-transformed control cassava plants digested with *Hind*III (A) and *Xba*I (B), and hybridized with SLCMV PCR-DIG labelled probe generated from plasmid DNA pEKH2IN2SLCMV and labelled by PCR using DIG-2’-deoxyuridine 5’-triphosphate (DIG-dUTP). (C) Accumulation of mRNAs in transgenic cassava lines; the steady-state transcript levels in most of the lines are low due to the specific degradation of the transgene product to siRNA soon after production. Lane M, molecular size marker. Lane WT, non-transformed wild-type plant. Lanes K3–K10, independent transgenic cassava lines.

Somatic embryos were inoculated with *Agrobacterium* and plants were regenerated as described above. In order to avoid possible chimeric plants, the shoots produced were subcultured in selective rooting medium. After 3 rounds of subculture, the chimeric shoots completely bleached and died or produced shoots on the surface of the medium while only 10 produced roots in the medium. Eight plantlets out of the 10 rooted shoots were transgenic as the 527 bp fragment of the *SLCMV* transgene (Fig. 4 A) and 700 bp fragment of *nptII* gene (Fig. 4 B) were amplified in the plants, producing a transformation frequency of 16% from the 50 somatic embryos that were inoculated. To validate the stable integration of the SLCMV transgene in transgenic plants, transgenic lines were characterized by Southern blotting. Genomic DNA was digested with *Hin*dIII, which cuts the T-DNA twice (at the 5′end of 35 S promoter and at the 5′end of *nos*-T terminator, see Fig. 3) producing a single copy of the transgene. As shown in Fig. 5A, the expected hybridization signal was obtained in all the transgenic plants tested except for transgenic line K5 and the wild-type plant. To determine the copy number of the transgene, transgenic lines showing integration of the insert were selected and digested with *Xba*I, which cuts the T-DNA once. The result presented in Fig. 5B shows 5 lines with a single locus and 1 with 2 loci. Expression pattern of *SLCMV* transgene in the transgenic plants was analyzed by northern hybridization by hybridizing total RNA with a DNA probe of *SLCMV* transgene. Our result shows that exception of line K3, which had high level expression, very little or no transgene transcript could be detected in the other transgenic lines tested. Since northern blots represent the steady state of the RNA concentration, but not the transcription rate; this could indicate that in these lines, the dsRNA became quickly degraded due to activation of PTGS [18]. No expression could be detected in the wild-type plants (Fig. 5C).

#### Plant inoculation and symptom screening

**Fig 6:**
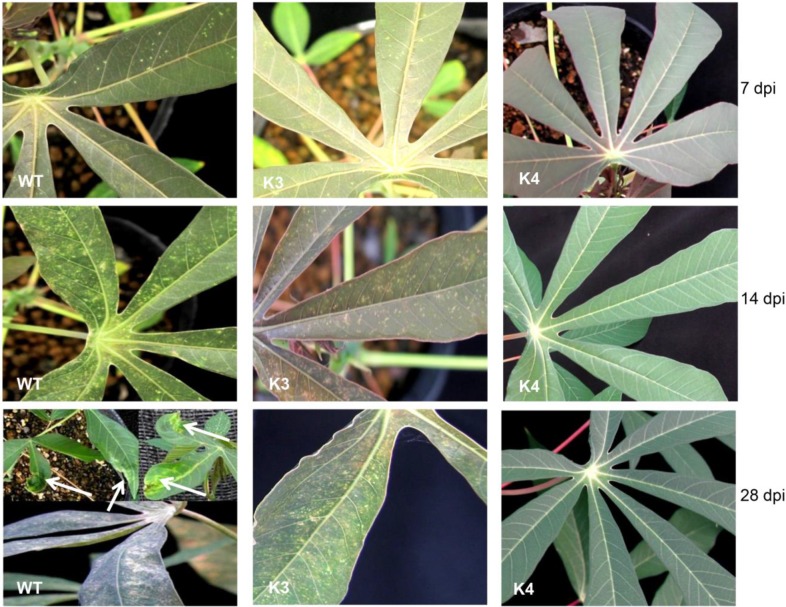
Screening of transgenic cassava lines for resistance to Sri Lankan cassava mosaic virus. Four week-old hardened transgenic and wild-type plants lines were mechanically inoculated twice with SLCMV. Symptom development was assessed every alternate day and photographs were taken at 7, 14 and 28 days post inoculation (dpi). WT, non-transformed wild-type plant. K3, susceptible transgenic line. K4 resistant transgenic line. Arrow indicates leaf deformation.

After screening transgenic plants by Southern and northern hybridization for siRNA accumulation and phenotype comparison of *in vitro* plants, we selected plant lines that were positive for agroinoculation with infectious clone of SLCMV. Since the number of plants was few, six lines, 3 plants per line were used for inoculation. Except for transgenic line K3 which was susceptible to the virus, all other transformants displayed high levels of resistance (Table 1). Upon inoculation, mild mosaic symptoms began to appear on new emerging young leaves of the wild-type plants and transgenic line K3, 7 dpi (Fig. 6, Table 1). The symptoms, which started as reduced chlorosis mostly close to the veins, quickly spread to the entire leaf and by 15 dpi, all the older leaves of the wild-type plant and most of the older leaves of line K3 have developed severe mosaic symptoms (Fig. 6). By 28 dpi, severe mosaic symptoms which included mosaic bleaching of leaves, leaf deformation and decrease in leaf size was observed in the control plant and transgenic line K3, whereas all the other transgenic lines (K4, K6, K7, K9 and K10) were free of symptom (Fig. 6). However, 40 days after inoculation, mild symptoms like reduced chlorosis, but not mosaic formation, leaf deformation or decrease in leaf size were observed in these transgenic lines (Table 1). These mild symptoms were completely obliterated 2 weeks later. This was further supported by the fact that the newly developed symptom-free recovered leaves had no viral-DNA accumulation as determined by PCR and RT-PCR compared with the symptomatic leaves of the wild-type and K3 transgenic plants. These observations clearly established that these transgenic lines displayed high levels of resistance to SLCMV.

**Table 1:** Sri Lankan cassava mosaic virus (SLCMV) symptoms severity score by visual detection in wild-type and transgenic plants.

Plant line tested^a^	Days taken for symptoms to appear	Symptom severity score /no. of leaves per grade^b^				No. of leaves with disease^a^ (%)	Disease index (%)^c^	Disease rating
		0	1	2	3			
WT	7	0	9	12	21	42/42 (100)	76.2	HS
MC	0	0	0	0	0	NA	NA	NA
K3	10	0	12	21	15	48/48 (100)	68.8	S
K4	45	63	3	0	0	3/66 (4.5)	1.5	HR
K6	43	45	6	0	0	6/51 (11.8)	3.9	HR
K7	45	48	3	0	0	3/51 (5.9)	2.0	HR
K9	42	39	9	0	0	9/48 (18.8)	6.3	HR
K10	42	54	6	0	0	6/60 (10)	3.3	HR

^a^Three plants were tested per transgenic line.

^b^Data represent the sum for the three plants tested and were recorded at 45 days post inoculation.

^c^Disease indices were calculated using the equation shown in materials and methods.

**Fig 7:**
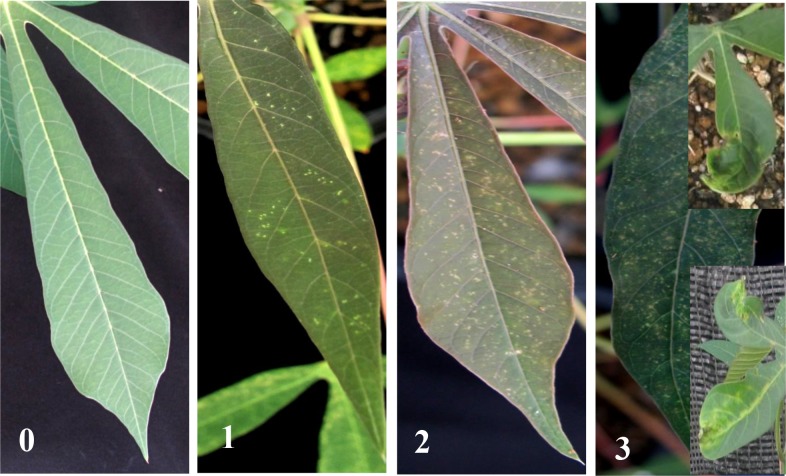
Representative leaves showing different degree of symptoms. These were used for evaluation of symptom severity score on a scale of 0–3 (0, no symptoms; 3, severe symptoms).

Disease rating is based on the calculated disease indices. Symptom severity score, the intensity of the disease on the leaf is expressed on a scale of 0–3 grades (0, no symptom; 1, mild/faint mosaic symptoms; 2, moderate mosaic symptoms; 3, severe mosaic symptoms, as shown in Fig. 7). Plants with a disease index of 0% were considered as immune, those with a disease index <25% as having high levels of resistance (HR), those with a disease index of 25.1–50.0% as being moderately resistant (MR), those with a disease index of 50.1–75.0% as susceptible (S), and those with a disease index of 75.1–100% as highly susceptible (HS), under the period of study. *NA* not applicable, *WT* wild-type plant, *MC* mock inoculated control plant, *K3-K10* transgenic cassava lines expressing hairpin SLCMV.

Since different leaves that emerged after inoculation usually showed a range of symptoms severity especially in the wild-type and susceptible transgenic line K3, we quantified disease severity per plant by evaluating the number of emerging leaves showing mosaic symptom at 45 dpi. Symptom severity was evaluated according to a standard scale of 0 (asymptomic) to 3 (severe mosaic, >76% of leaf area, leaf deformation) (Fig. 7). Based on the above scale, disease indices were calculated according to the formula shown in materials and methods.

As shown in Table 1, all the emerging new leaves of the wild-type plants and transgenic line K3 developed a ranged of symptoms, from mild (grade 1) to severe (grade 3). In the wild-type plants, of the 42 leaves that were scored for the 3 plants, 21 had severe symptoms classified to be of grade 3, 12 leaves were classified to be of grade 2, while 9 were classified to be of grade 1 severity score (Table 1). Consequently, disease index in this plant was high (76.2%) and the plant was rated as being highly susceptible to the virus (Table 1). In transgenic line K3, among 48 leaves scored, 15 were found to present symptoms of grade 3 severity score, whereas 21 and 12 leaves had grade 2 and 1 symptoms, respectively. Disease index calculated for this line was 68.8%, hence the line was classified as being susceptible (S) to the virus. It is worth noting that disease severity in these plants increased with age of the leaves i.e. lower leaves had symptoms which were mostly of grade 3 severity score compared to upper leaves in which the symptoms were of grade 2 or 1. Compared to the wild-type and transgenic line K3, only a few leaves of the remainder of the transgenic lines presented mild symptoms classified to be of grade 1 severity score (Table 1). For example, in transgenic line K4, only 3 leaves out of the 63 leaves that were scored from the three plants had symptoms classified to be of grade 1. In these transgenic lines, disease indices were very low ranging from 1.5% in line K4 to 6.3% in line K9. Therefore, these transgenic lines were rated as having high levels of resistance (HR) to the virus.

**Fig 8:**
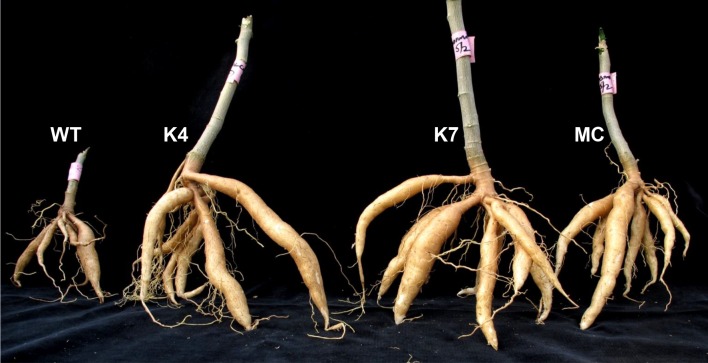
Cassava tubers harvested form plants inoculated with SLCMV. The tubers were harvested 60 days after inoculation. WT, non-transformed wild-type plant. K4 and K7 transgenic cassava lines expressing hairpin SLCMV AV2::AV1. MC, mock inoculated control plant.

Next we determined whether agronomic performance of these plants might be affected in the presence of the virus. First, we compared the plant height of the inoculated transgenic plants and the mock inoculated plant at 60 dpi (Table 2). There were no differences between the resistant transgenic lines and the mock inoculated control plant. In the mock inoculated plant, the plant height was 100.3 cm whereas in the resistant transgenic lines, the height ranged between 96.7 cm in line K9 to 102.1 cm in line K4 (Table 2). Compared to the mock inoculated control plant, the wild-type plant and the susceptible transgenic line K3 were significantly shorter (less than 60 cm). Second, we measured the yield of the plants which is an important aspect for biofuel industries. After taking the heights, the plants were uprooted and the number, length and weight of tubers were recorded. As shown in Fig. 8 and Table 2, the number of tubers in the wild-type and susceptible transgenic line K3 were significantly fewer (4 and 2, respectively) compared to the mock inoculated control plant and the resistant transgenic lines in which the number of tubers harvested per plant was 7. In fact, in line K9, up to 9 tubers were harvested per plant. Furthermore, there were no differences in length of tubers between the mock control plant and the resistant transgenic lines. However, in the weight of tubers, significant differences were observed. Tubers obtained from transgenic line K4 were noted to weigh more than those harvested from the other resistant transgenic lines and the mock inoculated control plant (Table 2). Together, these results show that agronomic performance of the resistant transgenic lines was unimpaired after infection with the virus.

**Table 2:** Plant height and yield characteristics in Sri Lankan cassava mosaic virus (SLCMV) infected transgenic and wild-type plants 60 days post inoculation.

Plant line tested	No. of leaves scored	No. of leaves with disease (%)	Plant height (cm)	Number of tubers	Average length of tuber (cm)	Weight of tuber (g/FW)
WT	16	16/16 (100)	59.5	4	6.3 ± 0.1a	13.6 ± 0.7b
MC	23	NA	100.3	7	10.7 ± 0.3b	56.4 ± 1.1c
K3	18	18/18 (100)	57.5	2	5.7 ± 0.1a	8.42 ± 0.5a
K4	25	0/25 (0)	102.1	7	10.8 ± 0.4b	65.4 ± 1.2d
K6	20	0/22 (0)	97.4	7	10.2 ± 0.2b	58.8 ± 1.3c
K7	19	0/19 (0)	99.4	9	10.5 ± 0.2b	59.5 ± 1.6c
K9	18	0/18 (0)	96.7	7	10.3 ± 0.3b	57.9 ± 1.1c
K10	23	0/23 (0)	98.3	6	10.4 ± 0.4b	58.4 ± 1.9c

Means with the same case letter in a given vertical array indicate no significant difference at 5% probability level using least significant difference (LSD) test. *WT* wild-type plant, *MC* mock inoculated control plant, *K3-K10* transgenic cassava lines expressing hairpin SLCMV. *NA* not applicable

We assessed the level of transmission of SLCMV to cassava by mechanical inoculation with infectious clone. Six out of the 7 Wild-type plants inoculated presented symptoms, which ranged from leaf curling, stunted growth and chlorosis, showing 85.7 percent transmission across two independent experiments (Table 3). High transmission efficiency of 71.4% was observed in the susceptible transgenic line K3; however, this line did not present the leaf curling symptom noted in the wild type plants. In the other transgenic lines, K4-K10, because of their high levels of resistance to the virus, the efficiency of transmission was very low, 14–28% (Table 3).

**Table 3:** Infectivity of SLCMV in transgenic and wild type cassava plants using mechanical inoculation with infectious clone.

Plant line	Plants infected/inoculated		Plants infected (%)	Symptoms
	Expt.1	Expt.2		
WT	3/3	3/4	85.7	Severe stunting, leaf curl, chlorosis
K3	2/3	3/4	71.4	Severe stunting, chlorosis
K4	1/3	0/4	14.3	Faint mosaic on leaf
K6	1/3	0/4	14.3	Faint mosaic on leaf
K7	1/3	0/4	14.3	Faint mosaic on leaf
K9	2/3	0/4	28.6	Faint mosaic on leaf
K10	1/3	0/4	14.3	Faint mosaic on leaf

#### Accumulation of siRNA in transgenic plants

**Fig 9:**
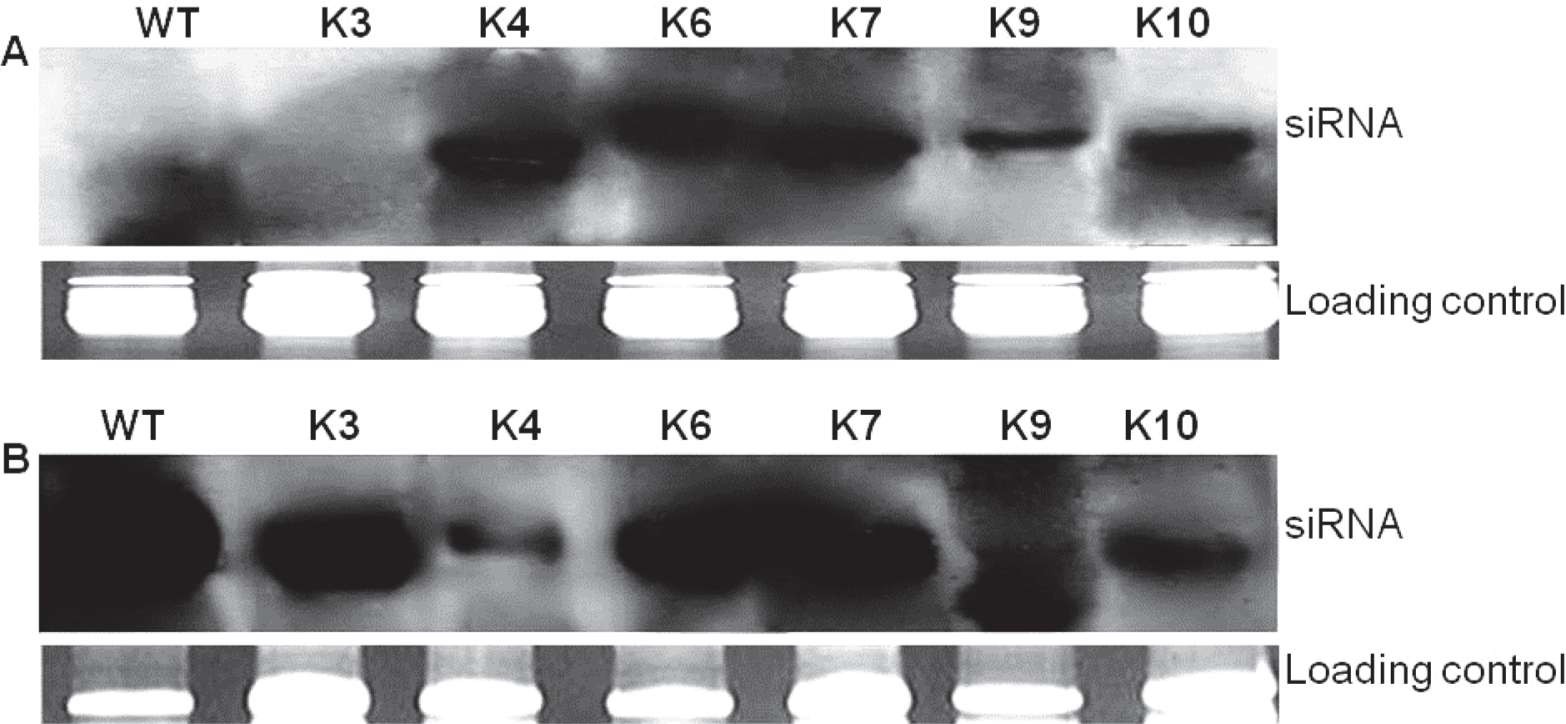
Detection of SLCMV specific siRNA in cassava plants before (A) and after (B) SLCMV inoculation. The inoculated plants were analyzed for siRNA accumulation at 60 days post inoculation (dpi). The lower panel shows the loading level of each sample after ethidium bromide staining. WT, Inoculated wild-type plant. K3-K10, transgenic lines expressing hairpin SLCMV AV2::AV1.

Before challenging the plants with SLCMV clone pSL7, northern analysis was carried out to detect SLCMV-specific siRNAs in transgenic and wild-type plants. The presence of siRNA is characteristic of PTGS and would indicate whether the PTGS response had been activated, especially in lines K4-K10 where transcript levels of the hairpin were either low or not detected. First, we screened the plants by PCR and RT-PCR to exclude a potential latent infection with SLCMV, which might result in virus-derived siRNA that are not of transgene origin. No SLCMV signal was detected in any of the lines tested (data not shown), indicating that no pre-induced PTGS effects are active in the transgenic plants. When northern blot was performed in the uninoculated transgenic plants, all transgenic lines, except line K3 were found to accumulate siRNAs signals of about 21–23 nt, homologous to the sense and antisense SLCMV sequences (Fig. 9A). No siRNAs were detected in the wild-type plants (Fig. 9A). Next, we probed for the presence of siRNA in uninoculated new leaves of transgenic and wild-type plants 60 days after challenge with SLCMV. All transgenic lines tested showed detectable levels of siRNA signals even in the susceptible transgenic line K3. Also, highly intense siRNAs signals were detected in the wild-type plant (Fig. 9 B). This result suggests that plant virus infection causes accumulation of siRNA in both susceptible and resistant phenotypes [17, 18]. The presence of SLCMV siRNA in the resistant transgenic cassava lines after infection can be interpreted as a natural antiviral defense responses, that is, however, overcome by the virus in the wild-type plant and transgenic line K3.

#### Screening of transgenic plants for presence of virus

**Fig 10:**
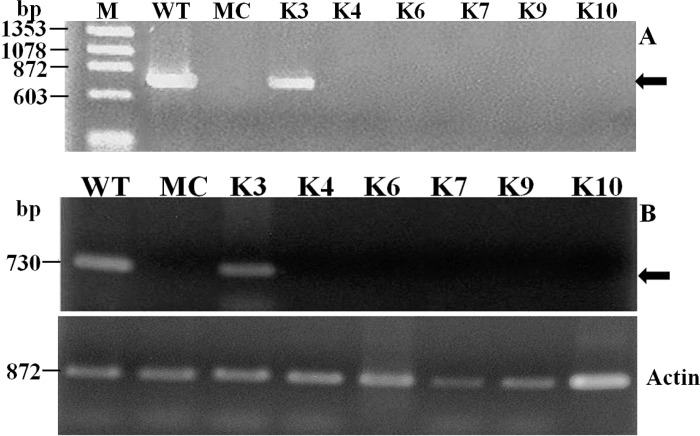
Screening of transgenic plants for detection of viral load in plants inoculated with SLCMV. PCR amplification (A) and RT-PCR analysis (B) of a fragment of SLCMV (DNA-A) in emerging new leaves of transgenic lines 60 days after infection with the virus. The lower panel represents rice actin (RAc1) gene used as an internal control for RNA input for RT-PCR.

PCR amplification (Fig. 10 A) and RT-PCR (Fig. 10B) were carried out to detect viral DNA 60 dpi in the same uninoculated new leaves analyzed for the presence of siRNA using coat protein specific primers. Viral DNA was not detected in new leaves of all the resistant transgenic lines (Fig. 10 A and B). On the contrary, strong signal of the CP gene were detected in new leaves of the inoculated wild-type plant and susceptible transgenic line K3 (Fig. 10 A and B).

## Discussion

Regeneration of whole plants from cell culture systems is often the limiting step in the application of various biotechnological techniques to crop improvement. A prerequisite for genetic modification of cassava is a reliable regeneration system. Establishment of a regeneration system for elite cassava cultivar such as KU50 will open up the possibility for genetic manipulation of the cultivar and provide the information needed for handling and introduction of useful genes. Adventitious shoot regeneration via somatic embryogenesis is highly desirable as the process affords high multiplication rates and results in propagules which possess both root and shoot axes. Somatic embryos may develop from single cell and, dependent on the genetic stability of the regeneration system, plants with novel genotypes may be recovered [33].

In several cassava cultivars, apical meristem and young leaf lobes have been frequently used for regeneration [33–36]. However, using these tissues is time consuming, often requiring several steps including cycling of somatic embryos, use of liquid medium and other plant growth regulators such as NAA to produce cotyledonary embryos and regenerated plants are obtained after several months of culture initiation. For example, in KU50, Saleim et al. [36] reported regeneration from apical and lateral buds; however, plantlets were only produced after several weeks of cycling and upon transfer of cotyledons to medium containing BA, IBA and NAA. In our study, we used axillary buds to regenerate cassava plants via somatic embryogenesis. Plant regeneration was achieved within 3 months of culture initiation and there was no need to induce and maintain FEC in GD. Rather, somatic embryos were subcultured and maintained in MSP and after transfer to MS2 supplemented with 0.5 mg/l BA and 0.5 mg/l IBA, cotyledons were induced which later produced shoots on hormone free MS medium, approximately 80% of the cotyledons produced shoots (Fig. 2). We optimized our protocol for the cultivar and used it to produce transgenic plants showing high levels of resistance to SLCMV. This rapid and efficient protocol could be useful for other cassava cultivars. However, as different cultivars appear to have different responses to different culture media and explants, optimal conditions need to be established separately for each cultivar if this protocol is to be used.

Cassava mosaic diseases can result in massive economic losses for industries using it for biofuel production, and also cause food and economic insecurity in countries where cassava is used as a staple food and cash crop. Therefore, attempts should be made to bring CMD under control in these regions. Unfortunately, control measures presently employed in infected regions are based on the chemical control of the vector population, with partial efficacy, environmental negative effect, elimination of natural enemies, and appearance of pesticide-resistant whitefly. Thus, producing disease resistant or tolerant varieties of cassava by genetic engineering is the best way to control the disease in the field. To date, considerable effort has been made by scientists to engineer cassava for resistance to African cassava mosaic virus by RNA silencing [13, 14, 35] using TMS60444. So far, such effort has not been applied to other cassava mosaic begomoviruses such as Sri Lanka cassava mosaic virus, and one of the most important cassava cultivar such as KU50 has not been engineered for resistance to CMD. In this study, we hypothesized that expression of dsRNA of a fragment of SLCMV in KU50 would lead to sequence-specific degradation of target mRNA interfering with viral replication and would reduce or prevent viral DNA accumulation and, consequently, appearance of symptoms. To achieve this, we produced a construct containing inverted repeat of 527 bp fragment of SLCMV. The DNA fragment contains a region encoding AV2 (137–493 bp) and half AV1 (297–527 bp). Among geminiviruses, amino acid sequences of AV1 (coat protein, CP) are fairly conserved [37] and known to be involved in the efficiency of virus infection. AV1 protein shares the same domain with AV2 (pre-coat protein), which together, they form a viral particle containing the sense fragment of Geminivirus DNA A. A recent report by Bull et al. [38] showed that AV2 of *East African cassava mosaic Zanzibar virus* (EACMZV) was necessary for infection in tobacco and cassava. The AV2 of *Indian cassava mosaic virus* (ICMV) has been shown to be involved in the cell-to-cell movement [39]. Furthermore, the AV2 protein of *tomato leaf curl virus* (ToLCV) and *tomato yellow leaf curl virus* (TYLCV) is shown to act as a potent suppressor of gene silencing activity. Silencing of *AV2* gene of ToLCV by antisense RNA [40] and *AV1/AV2* genes of ToLCV by artificial micro RNA [41] resulted to increased resistance to the virus in transgenic tobacco and tomato, respectively. Moreover, SLCMV DNA A has been reported to have properties of a monopartite begomovirus, which in the absence of DNA B, can induce upward leaf roll and vein swelling symptoms similar to those produced by monopatrtite begomovirus [10], indicating that the AV2 protein maybe involved in cell-to-cell movement. Therefore, we hypothesized that silencing of the *AV1*/*AV2* genes would disrupt the encapsidation and the next round of virus infection, and would be a useful strategy to develop broad-spectrum resistance against CMD in cassava, and other begomoviruses where numerous distinct virus species/strain may cause disease in a particular crop. Using this construct, we demonstrated that transgenic cassava expressing 527 bp fragment of SLCMV in the form of an intermolecular intron-hairpin RNA displayed high levels of resistance to SLCMV infection compared to the wild-type plant. We show that resistance was acquired by RNA silencing through the production of transgene-specific siRNA. The present study and work reported elsewhere [40, 41] suggests that *AV1*/*AV2* could be targeted to confer resistance to geminiviruses.

The use of siRNAs, an intermediate in the gene-silencing pathway, has become a powerful tool for specifically down regulating gene expression, and has been demonstrated successfully in a wide variety of cells and organisms [42]. It is one of the most important characteristics of RNA silencing and can be a reliable molecular marker that is closely associated with viral resistance in transgenic plants [18, 21]. In this work, before transgenic plants were analyzed for siRNA accumulation, we analyzed the plants by PCR and RT-PCR to exclude a potential latent infection with SLCMV, which might result in virus-derived siRNA that are not of transgene origin. The absence of viral load in the transgenic and wild-type plants confirmed the absence of siRNA which in turn confirmed the absence of premeditated PTGS process. Of the transgenic lines analyzed for siRNA prior to challenge by the virus, one line, K3, did not produce detectable levels of siRNA and was susceptible to the virus. This indicates that RNAi mechanism was not activated in this line; hence mRNA was not degraded to siRNA. Although we did not check partial transgene deletion or rearrangement, their effect on the absence of siRNA accumulation in line K3 cannot be ruled out. It has been shown that the presence of siRNA prior to challenge by virus plays a vital role in the resistance of transgenic lines to the virus [18]. Plants that produced detectable levels of siRNA prior to inoculation were resistant to viral infections, whereas those in which siRNA could not be detected were susceptible. When cassava lines were challenged with the virus, we observed accumulation of SLCMV siRNA signals in all transgenic lines tested including the susceptible line K3 and the wild-type plants. Indeed, the signals were more intense in the wild-type plants and in line K3 than in the other transgenic lines. In the wild-type plant and line K3 in which siRNA signals were detected but the plants were susceptible to SLCMV infections, it is most likely that the virus was actively replicating despite PTGS, whereas in the transgenic plants, the dsRNA transgene provides an additional defence mechanism leading to plant recovery. In the susceptible plants, there seem to be a fragile balance between the plant and the virus: the plant suppressing the virus via PTGS (and possibly other mechanisms) and the virus responds by rapid replication and suppression of the host’s silencing mechanism [43, 44]. To overcome the plant defence mechanism, certain plant viruses encode proteins that suppress RNA silencing. In Sri Lankan cassava mosaic virus, AC4 proteins have been reported to block post-transcriptional gene silencing [45]. It is possible that AC4 proteins inhibited gene silencing by sequestrating siRNAs and blocking them from entering RISC, thereby preventing their use as a guide for the recognition and sequence-specific degradation of homologous mRNAs. AC4 probably suppresses the antiviral defense system in plants at the beginning of infection, resulting to plants developing symptoms rapidly after inoculation [46]. We observed different levels of siRNA accumulation in the transgenic lines before and after virus challenge. The reason for the variability remains to be elucidated. However, it had been demonstrated that the variability in siRNA accumulation is reminiscent of the variability when antisense genes had been introduced for gene suppression [47]. The different response of resistant transgenic plants to virus infection does not seem to depend on the accumulation of siRNA. For example, line K4, Fig. 9, the most resistant line, had low siRNA accumulation after virus challenge. Kalantidis et al. [18] reported that CMV-specific short RNAs increases with the copy number of the transgene. However, in our study, with the exception of line K6 which had 2 copies of the transgene, all the other transgenic lines had one copy each. Therefore, we do not know whether the production of short RNAs is coupled to a particular locus or influenced by environmental factors. The developmental stage of a leaf at the time of siRNA analysis may also play a role in the variability. More studies are required to clarify these assertions.

Apart from transgenic line K3, which was susceptible to the virus (Table 1), the remainder of the tested lines developed symptoms in a few leaves at a much reduced level of severity (grade 1) with delayed appearance (more than 40 dpi). However, the plants quickly recovered as the symptoms completely disappeared 2 weeks later. Cassava plants can recover from mild symptoms but still carry virus load, which may be carried from one crop cycle to the next through the cuttings (stems) used as planting material since cassava is vegetatively propagated, or may be a source of inoculums by whiteflies. The propagation of plants that display no symptoms but carry a high virus load would be undesirable with regard to virus control under field conditions as such plants would serve as a source of inoculums for subsequent dissemination via whiteflies [48]. To confirm that the resistant lines do not contain virus load, they were analyzed for detecting viral DNA by PCR and RT-PCR. As no virus DNA was detected in these plants, it is likely that infected transgenic plants would be a poor source for spreading of the virus by whiteflies under field conditions.

RNA-mediated resistance has advantages for environmental biosafety over protein mediated resistance as the potential risks of heterologous encapsidation and recombination of virus are diminished. The major drawback in RNA silencing is that it is usually highly sequence specific, and viruses having between 10–15% nucleotide diversity are mostly insensitive to the technology [49]. Sequence comparison of SLCMV used in this study and other begomoviruses show that SLCMV is more closely related to ICMV (DNA A, 84%; DNA B, 94% nucleotide identity) than African cassava mosaic virus (DNA A, 74%; DNA B, 47% nucleotide identity) [10]. Although we did not test our transgenic plants for resistance to other begomoviruses, it is most likely that the resistance gained here will be effective against other geminiviruses as the amino acid sequences of AV1 (coat protein, CP) are fairly conserved among geminiviruses. Furthermore, the resistance gained here will be more effective against ICMV than ACMV since the nucleotide sequence used for hairpin in the construct and the SLCMV strain used for infection share high homology with ICMV.

In cassava, up to 90% of its starch is stored in the tubers, which makes them suitable for biofuel production. Therefore, high tuber yield is an important factor for bioethanol production in cassava. When cassava is attacked by CMD, there is reduced tuber yield in terms of number and size, many of them rotting, making them unsuitable for use for biofuel production. In this regard, we performed another round of inoculation and checked the agronomic performance of the plants in the presence of the virus. It was encouraging to see that, besides the absence of disease symptoms and virus load in the resistant transgenic plants, the agronomic performance of these plants was not affected by infection with the virus and the expression of SLCMV dsRNA did not intrinsically cause yield depression, as the plants presented similar growth when compared to the mock inoculated control plants and revealed no apparent reduction in the amount of tubers produced as well as weight of tubers (Table 2). This desirable yield components coupled with high levels of resistance to SLCMV in these transgenic lines makes them better candidates for exploitation in the production of biomass as well as bioethanol.

In this study, since the number of replicates was low, we used mechanical inoculation with infectious clone to introduce SLCMV DNA into cassava. Cloned SLCMV DNA has been shown to be infectious to cassava by agroinoculation [50] and biolistic inoculation [11, 51]. Here, we show that mechanical inoculation can be used to introduce SLCMV DNA to cassava, with variable degrees of infectivity. The efficiency of transmission observed here was seen to be similar to that reported earlier for SLCMV by agroinoculation [49] and for cassava brown streak disease (CBSD) by graft inoculation [52]. Altogether, this method, in addition to agroinoculation and biolistic approach, would be useful in screening large collections of cassava germplasm for SLCMV resistance.

## Materials and Methods

### Production of SEs and regeneration

Commercially important cassava cultivar KU50, which was provided from Genetic Resource Unit of CIAT, Cali, Colombia, was tested for its regeneration ability. The plant material was maintained as shoot cultures on MS2 (MS salts and vitamins [53] supplemented with 2% sucrose and 2 μM CuSO_4_), solidified with 0.8% plant agar at 28°C with a 16-h photoperiod and subcultured at 8 week-intervals. All media supplements were added prior to adjusting the pH to 5.8 and autoclaving at 121°C for 15 min. For dark cultures, all plate cultures were sealed with parafilm and wrapped in aluminum foil. All cultures were maintained at a temperature of 28 ±2°C.

The regeneration protocol is according to the method described by Bull et al. [54], but with significant modification. Nodal stem cuttings measuring about 10 mm were obtained from *in vitro* grown plants maintained in MS2. The tissues, which contained tiny axillary bud at each node, were cultured onto plates containing MS2 supplemented with 10 mg/l BA (MSB) for axillary bud enlargement. Six days later, enlarged axillary buds were isolated and transferred to plates containing MS2 supplemented with 12 g/l picloram (MSP). After 2 weeks in culture, somatic embryo structures (SEs) were produced on the axillary buds. Once the SE developed to torpedo stage, they were transferred to the same fresh medium (MSP) at 2 weeks interval for 6 weeks (3 cycles) for further growth. At each transfer, the soft non embryogenic tissues were removed. Four weeks later, the somatic embryos were transferred to MS2 supplemented with 0.5 mg/l BA and 0.5 mg/IBA for cotyledon production. To produce shoots and roots, the cotyledons were transferred to MS2 medium but without 2 μM CuSO_4_ and exposed to light. Roots and shoots developed within 4 weeks in culture.

### Plant transformation

#### RNAi vector construction

A DNA fragment (527 bp) of SLCMV segment A (KC424490) was isolated by RT-PCR using a pair of Deng’s primers [55] (Table 4). The DNA fragment has the highest homology to Sri Lanka cassava mosaic virus isolate Adivaran, encoding AV2 (137–493 bp) and half (297–527 bp) of AV1. The PCR product was purified and cloned into Gateway entry vector, pCR8/GW/TOPO (Invitrogen, Life Technology, Tokyo), which contains *attL1* and *attL2* recombination sites. The transformants were subjected to sequencing the targeted DNA fragment by using M13 primers and analyzed. The correct transformant containing the 527 bp fragment was subcloned in the sense orientation between the attB2 and attB1 recombination sites and in the antisense orientation between the attB1 and attB2 recombination sites on either ends of a 278 bp fragment of cat1-intron in the binary vector pEKH2IN2 (Nakamura *et al*. unpublished) by eLR clonase (Invitrogen, New Zealand) recombination reaction. The product was transformed into TOP10 chemical competent cells (Invitrogen, New Zealand) and selected on kanamycin-containing LB plates. Clones were verified by digestion with *Eco*RV. The plasmid, which contains marker genes for neomycin phosphotransferase (nptII) and hygromycin phosphotransferase (hpt), and the SLCMV gene being driven by cauliflower mosaic virus (CaMV) 35S promoter, was introduced into *Agrobacterium tumefaciens* strain EHA105 by triparental mating.

**Table 4:** Primers used for amplification.

Primer	Primer sequence
Deng-5P	5'-TAATATTACCKGWKGVCCSC-3'
Deng-3P	5'-TGGACYDTRCAWGGBCCTTCACA-3'
SLCMV-5P	5'-ATGTCCCCCACTCAGAACGCTCCC-3'
SLCMV-3P	5'-CTAGGAACATCTGGGCTTTTGAAC-3'
NPTII-5P	5'-GATGTGATATCTCCACTGAC-3'
NPTII-3P	5'-CGCAAGACCGGCAACAGGAT-3'
SLCMV-A-5P	5'-ATGTCGAAGCGACCAGCAGATATC-3'
SLCMV-A-3P	5'-GCGTAGCGTATACAGGGTTAGAGG-3'
RAc1-5P	5'-GAAAATGGTGAAGGCTGGTTTTG-3'
RAc1-3P	5'-AGG ATTGATCCTCCGATCCAGA-3'.

#### Agrobacterium culture and plant transformation

*Agrobacterium tumefaciens* strain EHA105 carrying the binary vector pEKH2IN2SLCMV was cultured overnight on a reciprocal shaker (120 cycles/min) at 28°C in 50 ml liquid LB medium containing 50 mg/l kanamycin and 25 mg/l chloramphenicol. The bacterial suspension was centrifuged and then resuspended to final density of OD600 = 0.5 in inoculation medium, which was liquid MS2 containing 200 μM acetosyringone and incubated at room temperature for 1 h before inoculation.

For axillary bud enlargement, nodal stem cuttings were cultured on MSB for 6 days. Thereafter, the enlarged axillary buds were isolated and cultured on MSP for 6 weeks with subcultures at 2 week-intervals for somatic embryo (SE) induction. The SEs were cycled again in the same medium for 4 weeks before being used for infection with *Agrobacterium*. These tissues were transferred to Erlenmeyer flasks containing *Agrobacterium* suspension for inoculation. The flasks were wrapped with aluminum foil and incubated on rotary shaker (R-20 min) at 80 cycles/min for 30 min. The tissues were retrieved by filtering, and placed on filter papers to remove excess bacteria. The SEs were then cultured onto plates containing MSP supplemented with 200 μM acetosyringone. The plates were wrapped with aluminum foil and co-cultivated at 24°C for 2 days and then exposed to light for another 2 days. Thereafter, the SEs were transferred to the MSP containing 20 mg/l meropenem and maintained at 28°C, dark for 1 week (pre-selection). After 1 week, the SEs were cultured on MSP containing 50 mg/l kanamycin and 20 mg/l meropenem for 2 weeks for maturation. If bacteria overgrowth was noted after co-cultivation, the tissues were washed three times with sterile distilled water containing 10 mg/l meropenem. For production of cotyledons, SEs were transferred to MS2 supplemented with 0.5 mg/l BA and 0.5 mg/l IBA and containing the same concentrations of antibiotics and exposed to light. Cotyledons were transferred to MS2 but without 2 μM CuSO_4_ for shoot and root development. Regenerated plantlets having shoots and roots were excised and cultured in selective rooting medium, which was MS medium containing 3 g/l gelrite and 100 mg/l kanamycin. Putative transgenic shoots formed roots in the medium, while non-transgenic shoots developed roots on the surface of the medium. Therefore, shoots which produced roots in the medium were selected and micropropagated *in vitro* for subsequent molecular analysis and transfer to soil.

#### Molecular analysis of transgenic plants

Genomic DNAs were extracted from young leaves of *in vitro* plants using a modified cetyltrimethylammonium bromide (CTAB) protocol [56]. Total RNA was isolated using Guanidine thiocyanate protocol [57] with slight modification. PCR, Southern and Northern analyses were carried out following standard protocols [58]. PCR amplification for the SLCMV was performed using primers specific to the SLCMV (SLCMV-5P and SLCMV-3P), whereas for *npt*II gene, the primers used were NPTII-5P and NPTII-3P (Table 4). For Southern blot, 15μg of the DNA was digested overnight with either *Hin*dIII, which cuts the T-DNA at two positions or *Xba*I, which cuts the T-DNA once, separated on a 0.8% agarose gel, transferred to a nylon membrane (Immobilon-Ny+ Transfer Membrane; Millipore Co, Billerica, MA, USA) and probed with SLCMV PCR-DIG labelled probe. The probe DNA fragment was generated from plasmid DNA pEKH2IN2SLCMV and labelled by PCR using DIG-2’-deoxyuridine 5’-triphosphate (DIG-dUTP) in the sense and antisense direction according to the supplier’s instruction (PCR DIG probe synthesis kit, Roche Diagnostic GmbH, Boehringer Mannheim, Germany). Prehybridization (3 h) and hybridization (overnight) were carried out at 38°C and 42°C respectively, using high-SDS hybridization buffer containing 50% deionized formamide, 5 × SSC, 50 mM sodium phosphate (pH 7.0), 2% blocking solution, 0.1% N-lauroylsarcosine and 10% SDS. Post hybridization washes and detection using CDP star were performed according to the instruction manual of the DIG labelling and Detection System (Roche Diagnostics, Mannheim, Germany). For northern blot, 15 μg of the total RNA was denatured and separated on 1.5% agarose gel. Separated RNAs were transferred to nylon membrane, probed with a DNA probe of SLCMV gene and detected following the same procedure used in Southern blot analysis, with slight modification where necessary. Pre-hybridization (2 h), hybridization (overnight) and washing were performed at 50°C.

#### Detection of short RNAs in selected transgenic lines before and after virus challenge

Total RNA was isolated as described above, and small RNAs were enriched from the total RNA by polyethylene glycol (PEG; MW_8000_) as described by Smith and Eamens [59]. Thirty micrograms of small RNAs were electrophoresed on a 17% polyacrylamide gel containing 7 M urea and 10x TBE (Tris Borate EDTA). siRNAs were transferred to Immobilon-NY+ membrane (Millipore Corporation, Billerica, MA, USA) in a semi-dry cell (Semi-dry blotting apparatus NA-1512, Nippon Eido, Tokyo, Japan) for 1h at 10V/400mA and subjected to northern hybridization with a probe obtained by the *in vitro* transcription of the SLCMV gene in the antisense and sense orientation using T7 RNA polymerase from DIG RNA labelling Kit SP6/T7, Roche Diagnostic GmbH, Boehringer Mannheim, Germany. Briefly, 10 μg of plasmid DNA extracted from pCR8/GW/TOPO, was linearized by digesting with *Apa*I at 37°C for 3 h. The linearized plasmid was purified via phenol/chloroform extraction and ethanol precipitation. Then, 1 μg of the purified template DNA was mixed with 12 μl of DMPC water, 2 μl of 10x NTP labelling mix, 2 μl of 10x Transcription buffer, 1 μl of Protector RNase inhibitor and 2 μl of RNA polymerase T7. The mixture was incubated at 37°C for 2 h and 2 μl of 0.2 M EDTA (pH8.0) was added to stop the reaction. In order to improve the signal, the probe was hydrolyzed with carbonate buffer (60 mM sodium carbonate and 40 mM sodium bicarbonate), and incubating at 60°C for 5 h [60]. Prehybridization (30 min) and hybridization (overnight) were performed at 37°C. Posthybridization washes were performed with 2x SSC for 2 x 5 min at room temperature, followed by 0.1x SSC/0.2%SDS for 2 x 15 min at 50°C. siRNA signals were detected using CDP-star (Roche Applied Science), as described in the DIG system and DIG application manual (Roche Diagnostic GmbH, Boehringer Mannheim, Germany).

#### Plant inoculation with SLCMV and symptom evaluation

Four week-old hardened transgenic and wild-type plants lines obtained from 3 generations of *in vitro* cuttings were mechanically inoculated twice with SLCMV clone pSL7 and maintained in the greenhouse at 22 ± 2°C, 60% relative humidity. Six lines, 3 plants per line were used for the assay. First, two completely opened young leaves were rubbed with carborundum (abrasive agent) to create wounds. The leaves were rinsed with 50 mM sodium phosphate buffer containing 0.4% sodium sulphite, pH 7.5. Then, SLCMV clone pSL7 was applied by rubbing each leaf several times back and forth using a cotton bud. Second, three days after the first inoculation, another young leaf was cut off at the node. The cut surface on the stem was rinsed with sodium phosphate buffer and the same concentration of viral clone was applied by rubbing several times. In parallel, mock control plants were inoculated with 50 mM sodium phosphate buffer only. The experiment was performed twice.

Symptom development was assessed every alternate day but the final classification was recorded at 45 days post inoculation (dpi). The number of leaves per plant showing mosaic symptoms was recorded at 45 and 60 dpi. Also, the plant height, number, length and weight of tubers were recorded at 60 dpi. Disease symptom severity on fully expanded leaves was recorded at 45 dpi on a scale of 0–3 (0, no symptom; 1, mild chlorotic pattern over entire leaflets; 2, moderate mosaic pattern throughout the leaf, narrowing and distortion of the lower one-third of leaflets; class 3, severe mosaic, distortion of two thirds of the leaflets). Disease indices were calculated using the method described by [61] as follows:

$$\text{D}.\text{I}=\frac{\left(n_{1}+{2n}_{2}+{3n}_{3}\right)100}{3\left(n_{0}+n_{1}+2n_{2}+3n_{3}\right)}$$

Where n is the number of leaves in each grade (0–3) with respect to the symptom severity score. Plants with a disease index of 0% were considered as immune, those with a disease index <25% as having high levels of resistance (HR), those with a disease index of 25.1–50.0% as being moderately resistant (MR), those with a disease index of 50.1–75.0% as susceptible (S), and those with a disease index of 75.1–100% as highly susceptible (HS).

#### Determination of viral load on uninoculated new leaves by PCR and RT-PCR

Sixty days after infection, PCR and RT-PCR analyses were carried out to detect SLCMV cassava leaves. DNAs were extracted from new leaves that emerged from infected transgenic and wild-type plants using CTAB. Primers SLCMV-A-5P and SLCMV-A-3P (Table 4) were used to amplify a 700 bp fragment from SLCMV genome (DNA-A). For RT-PCR, total RNAs were extracted from new leaves that emerged from infected transgenic and wild-type plants using guanidine thiocyanate. The RNAs were treated with DNase and RT-PCR was performed using the Superscript III RNase H reverse transcriptase (RT) kit (Invitrogen). The cDNAs were then used as templates for the amplification of SLCMV-CP gene with the same pair of primers (SLCMV-CP-5P and SLCMV-CP-3P) used for PCR amplification. Also, PCR analysis of rice actin gene (RAc1; X16280) was performed as a control to check the quality of cDNA synthesized in the RT-PCRs using the primers RAc1-5P and RAc1-3P (Table 4).

### Statistical analysis

Data on yield (length and weight of tubers) were subjected to analysis of variance (ANOVA) test using a completely randomized design (CRD). Means were separated by least significant difference (LSD) test at the 5% probability level. All computations were performed using SPSS 17.0 statistical package for Windows (SPSS, Inc., Chicago, IL, USA).
